# Achilles Tendon Mechanical Behavior and Ankle Joint Function at the Walk-to-Run Transition

**DOI:** 10.3390/biology11060912

**Published:** 2022-06-14

**Authors:** Andrea Monte, Paolo Tecchio, Francesca Nardello, Paola Zamparo

**Affiliations:** 1 Department of Neurosciences, Biomedicine and Movement Sciences, University of Verona, 37124 Verona, Italy; andrea.monte@univr.it (A.M.); paolo.tecchio@ruhr-uni-bochum.de (P.T.); francesca.nardello@univr.it (F.N.); 2 Human Movement Science, Faculty of Sports Science, Ruhr University Bochum, 44801 Bochum, Germany

**Keywords:** joint function, locomotion, elastic energy, muscle–tendon behaviour

## Abstract

**Simple Summary:**

In this study we investigated the ankle joint functional indexes and the Achilles tendon mechanical behaviour (changes in AT force and power as a function of speed and gait) during walking and running at speeds close to transition speed (about 7.2–7.5 km/h in healthy adults), to better elucidate the mechanical determinants of the walk-to-run transition. Our results indicate that, when walking at speeds faster than the typical transition speed (7.5–8.5 km/h), the Achilles tendon mechanical behavior is impaired: the force acting along its line of action is reduced, as well as its contribution in determining the total mechanical power of the muscle–tendon unit. Moreover, our data suggest that the walk-to-run transition could be partially explained by the need to preserve the spring-like function of the ankle joint (which is indeed lower in walking than in running at speeds > 7.5 km/h).

**Abstract:**

Walking at speeds higher than transition speed is associated with a decrease in the plantar-flexor muscle fibres’ ability to produce force and, potentially, to an impaired behaviour of the muscle–tendon unit (MTU) elastic components. This study aimed to investigate the ankle joint functional indexes and the Achilles tendon mechanical behaviour (changes in AT force and power) to better elucidate the mechanical determinants of the walk-to-run transition. Kinematics, kinetic and ultrasound data of the gastrocnemius medialis (GM) were investigated during overground walking and running at speeds ranging from 5–9 km·h^−1^. AT and GM MTU force and power were calculated during the propulsive phase; the ankle joint function indexes (damper, strut, spring and motor) were obtained using a combination of kinetic and kinematic data. AT force was larger in running at speeds > 6.5 km/h. The contribution of AT to the total power provided by the GM MTU was significantly larger in running at speeds > 7.5 km/h. The spring and strut indexes of the ankle were significantly larger in running at speeds > 7.5 km/h. These data suggest that the walk-to-run transition could (at least partially) be explained by the need to preserve AT mechanical behaviour and the ankle spring function.

## 1. Introduction

The contractile elements (muscles) that power vertebrate locomotion are associated, within a given muscle–tendon unit, with deformable tissues (such as tendons) which stretch and store elastic strain energy when force is applied to them and recoil (shorten) to release the energy when this force is released [1,2,3]. In human locomotion, the elastic tissues serve a diverse set of functions, including metabolic energy conservation, amplification of muscle power output and attenuation of muscle power input [2].

A lingering question in the biomechanical community regards the mechanisms behind the transition from walking to running. From a physiological point of view, gait transition is triggered by metabolic energy expenditure at the whole-body level [4]: above a certain walking speed it is metabolically cheaper to run than to walk. Furthermore, it was observed that spontaneous transition speed is associated with a decrease in plantar flexor muscle fibres’ ability to produce force [5,6,7] and a reduction of the gastrocnemius medialis force contraction capacity [8]. This body of evidence reinforces the idea that the determinants of the walk-to-run transition could be related to mechanical alteration at the ankle level in terms of the contractile capacity of plantar-flexor muscles. Potentially, however, the elastic components of the plantar-flexor muscle–tendon unit could also play a role in triggering the transition.

Among the elastic tissues of the human body, the Achilles tendon (AT) is considered one of the key evolutionary advantages for human locomotion [9]. Indeed, during walking and running the contribution of AT elastic strain energy to the positive work generated by the triceps surae muscle–tendon units (MTUs) is larger than 50% [5,10]. Furthermore, during walking and running the plantar flexors MTUs generate more than 40% of the total mechanical work in the whole-body [11]. In addition, Farris and Sawicki [5] showed that the series-elastic elements of the gastrocnemius medialis released approximately 55% of the total mechanical power generated by the MTU during walking at increasing speeds and that switching to running allows for an increase in their contribution. However, to our knowledge, so far no studies have investigated the possible role of the Achilles tendon mechanical behavior in determining the walk-to-run transition.

Regarding the ankle joint, its mechanical behaviour can be investigated using a novel approach proposed by Qiao and Jindrich [12]. They characterised joint function into four distinct behaviours: (1) a motor that generates positive work; (2) a spring that stores and releases elastic strain energy; (3) a strut that generates high force with small length changes; and (4) a damper that lengthens to absorb energy. This classification could be useful in understanding how joints change their function to properly satisfy various mechanical demands [13,14,15].

This approach, in combination with the investigation of the Achilles tendon mechanical behaviour, could thus provide further insight into the determinants of the walk-to-run transition. To the best of our knowledge, the joint functional role was investigated in walking and running only at a given speed (1.4 and 4 m/s, respectively: [14])

Hence, the aim of this study was to investigate the ankle joint functional indexes and the Achilles tendon mechanical behaviour (force and power) during walking and running at speeds close to the transition speed (e.g., around 7.2–7.5 km/h in healthy adults), to better elucidate the mechanical determinants of the walk-to-run transition. We hypothesised that the transition occurs, among other things, to preserve the spring-like function of the ankle and the elastic energy recoil of the Achilles tendon.

We hypothesised an impairment in the force applied to the Achilles tendon at high walking speeds leading to a reduction in the AT mechanical function. Were this hypothesis true, we should also observe a decrease in the spring index at the ankle level as well as a decrease in the AT contribution to the MTU mechanical power at walking speeds faster than the transition speed.

## 2. Materials and Methods

### 2.1. Participants

Ten healthy participants (5 females and 5 males; age: 28.5 ± 5.3 years; height: 1.70 ± 0.05 m; mass: 65.2 ± 7.2 kg; lower limb length: 840.9 ± 25.7 mm; leg length: 410.5 ± 19.2 mm) were recruited for this study. The subjects were moderately active and practiced mainly recreational running (2 sessions per week). The study agreed with the Declaration of Helsinki for studies on human subjects. The participants did not report any recent history of lower limb neuro-musculoskeletal injury in the last 24 months. The local ethical committee approved the experimental protocol (protocol number 2020-UNVRCLE-161 0142370) and all subjects gave their written informed consent.

### 2.2. Experimental Design

Before the test, the participants familiarized themselves with the devices and the experimental procedures. During the test, kinematics of the body segments and kinetic and ultrasound data of the gastrocnemius medialis muscle–tendon junction were investigated at different speeds (from 5–9 km·h^−1^) during overground walking and running.

### 2.3. Protocol

The participants were asked to walk and run barefoot using their self-selected walking and running technique over the entire velocity range; they had ~10 m to reach and maintain the desired speed before stepping over a force platform. Locomotor velocity was controlled (by means of a stopwatch) by an external operator that gave feedback about the velocity; the participants were then able to adjust the locomotor velocity for the following tests.

### 2.4. Data Collection

During each trial, a 3D motion capture system (8 cameras; Vicon, Oxford, UK, sampling at 200 Hz) was used to record the three-dimensional trajectories of 34 markers (lower-body modified Plug-in Gait). Ground reaction forces (GRF) were recorded using a force platform (AMTI, Watertown, MA, USA, sampling at 1000 Hz). A B-mode ultrasound scanner (Telemed MicrUs EXT-1H rev.D, Vilnius, Lithuania) was used to record images (sampling rate = 48 Hz) with a depth and width of 40 and 60 mm, respectively. The ultrasound probe was firmly attached to the right leg and used to detect the muscle–tendon junction (MTJ) displacement of the gastrocnemius medialis (GM) muscle–tendon unit (MTU). The transducer position was tracked by the motion capture system using markers that identified a 3D printed custom probe case in the 3D space permitting us to synchronize ultrasound imaging with motion capture analysis. Ultrasound, kinematic and kinetic data were synchronized by a digital output generated by the ultrasound scanner that triggered all instrumentation.

During the trials, the Achilles tendon curvature was taken into account as described by Tecchio et al. [16] using 6 markers along the Achilles tendon line of action. This marker configuration allows for a better representation of the Achilles tendon moment arm and mechanical behavior compared to other marker-sets (e.g., straight line).

### 2.5. Data Analysis

We recorded about 480 good trials at walking/running speeds ranging from 5 to 9 km/h. A trial was considered “good” when the entire foot impacted the force platform and when the forward velocity of the body was constant 2 m before and 2 m after impact (measured a posteriori from kinematic data). We then performed a further selection to obtain 4 clusters of walking/running speeds: 5.5 ± 0.20, 6.5 ± 0.20, 7.5 ± 0.20, 8.5 ± 0.20 km/h (including at least 30 trials each). The trials outside these clusters were disregarded to avoid an overlapping of speeds between clusters (a possible confounding factor). To check that the participants were walking or running at these speeds we calculated the duty factor (DF) as the ratio between the contact and the stride time (expressed in %): when DF > 50% the subjects are walking, when <50% they are running (e.g., [3,17]).

We then estimated the walk-to-run transition speed for each subject as: v = $\sqrt{0.5\cdot g\cdot L}$; where 0.5 is the Froude number that corresponds to the transition speed, *g* is the gravitational acceleration and *L* the leg length (e.g., [3,17]).

Markers trajectories and kinetic data were filtered with a fourth and second-order low pass and zero-lag Butterworth filter with a cut-off frequency of 12 and 15 Hz, respectively. The stance phase was identified from the GRFs with a threshold of 1 N. Ankle joint angle was calculated using an inverse kinematics approach, while net ankle moment was calculated using a 3D inverse dynamics method [18,19].

The gastrocnemius medialis MTU length was calculated according to the Hawking and Hull equation [20], by knowing the ankle and the knee angles during the stance phase. AT length was calculated by taking into account AT curvature (e.g., [16]) and the MTJ displacement was tracked manually frame by frame (Tracker, Physlets.org). At the end of these procedures, the GM MTU and the Achilles tendon length were obtained frame by frame during the entire stance phase.

During the stance phase, the AT internal moment arm (IMA) was calculated in the sagittal plane, taking into account its curvature (e.g., [16]). AT IMA was defined as the minimal distance between the ankle joint center of rotation (identified with a marker positioned on the lateral malleolus) and the AT line of action, frame by frame. AT force was then estimated by dividing net ankle moment by the AT IMA, frame by frame. The force applied at the MTU level is considered equal to that applied at the tendon level (e.g., [5,19]).

**Figure 1:**
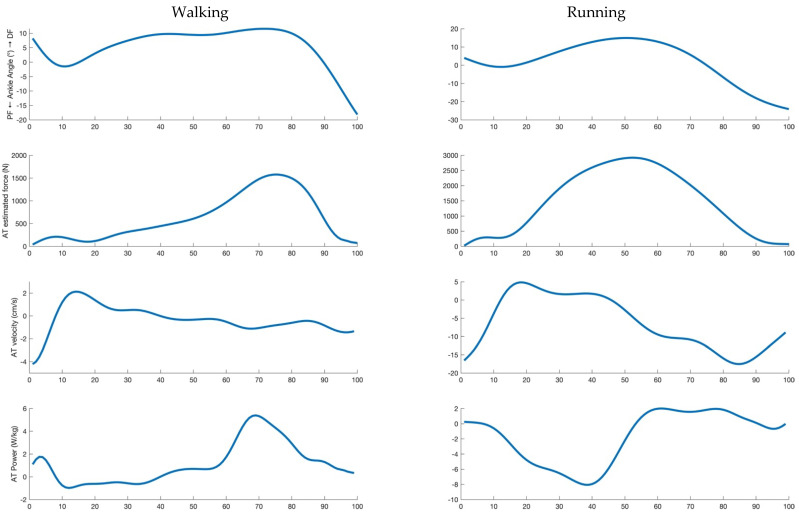
From top to bottom: ankle angle, Achilles tendon force, Achilles tendon velocity and Achilles tendon power during the stance phase of walking (panels on the **left**) and running (panels on the **right**) for a representative subject at a speed of about 7.5 km/h.

AT and GM MTU velocity were calculated as the first derivative of their length during the entire stance phase. AT and GM MTU mechanical power were calculated as the product of force (assumed to be the same for AT and the MTU) and the corresponding velocities (which are different) during the entire stance phase. An example of the investigated parameters is reported in Figure 1 for a representative subject. The mean values of AT and MTU force, power and velocity data were calculated in the propulsive phase only (positive values of the antero-posterior force until take-off). The percentage of the total MTU mechanical power provided by AT was finally calculated (propulsive phase only).

Joint functional indexes were calculated (during the entire stance phase) in accordance with Qiao and Jindrich, [12]; each index was calculated relative to each other, in order to obtain a cumulative percentage of 100%. The ‘‘primary” functional role of a joint is then determined by the functional index with the greatest percentage. The strut index was calculated as:

$$strut\;index=max(1-\frac{\left(tTD-tTO\right)\int_{tTD}^{tTO}\left|\left.Pjoint\right|\right.dt}{\int_{tTD}^{tTO}\left|\left.Mjoint\right|\right.dt},\;0)\times 100\%$$

where *tTD* and *tTO* are the time of touch-down and take-off, respectively, and *Pjoint* and *Mjoint* are the power and moment generated, respectively, at the joint level. This index expresses the proportion of joint moment that contributes to work production. Indeed, joints with large moments and low angular displacements or energy fluctuations act as a strut reaching strut index values close to 100%.

Spring-like function at a joint level is characterised by two phases: energy absorption (e.g., joint flexion) and energy release (e.g., joint extension). The spring index was then calculated as:

$$\begin{array}{l} spring\;index=\frac{2\cdot \min\;\left(\left|\left.W^{-}\;flexion\right|\right.,\;\left|\left.W^{+}\;extension\right|\right.\right)}{\left|\left.W^{-}\;flexion\right|\right.\;+\;\left|\left.W^{+}\;extension\right|\right.}\times 100\%\\ \;\;\;\;\;\;-\;strut\;index \end{array}$$

where *W^−^ flexion* represents the negative work during the absorption phase, and *W^+^ extension* the positive work during the propulsion phase. Motor-like function describes the work produced during the entire contact time (i.e., stance phase) without the contribution of the elastic energy sources. The motor index was then calculated as:

(1) $$\begin{array}{l} motor\;index=\frac{\left|\left.W^{+}\;stance\right|\right.\;-\;\min\;\left(\left|\left.W^{-}\;flexion\right|\right.,\;\left|\left.W^{+}\;extension\right|\right.\right)}{\left|\left.W^{-}\;stance\right|\right.\;+\;\left|\left.W^{+}\;stance\right|\right.}\times 100\%\\ \;\;\;\;\;\;-\;strut\;index \end{array}$$

where *W^+^ stance* and *W^−^ stance* are the integral of the positive and negative mechanical power generated during the entire contact time, respectively. Finally, the damper-like function describes the energy dissipated during the absorption phase, without the contribution of the potentially spring-like characteristics. This index was calculated as:

(2) $$\begin{array}{l} damper\;index=\frac{\left|\left.W^{-}\;stance\right|\right.\;-\;\min\;\left(\left|\left.W^{-}\;flexion\right|\right.,\;\left|\left.W^{+}\;extension\right|\right.\right)}{\left|\left.W^{-}\;stance\right|\right.\;+\;\left|\left.W^{+}\;stance\right|\right.\;}\\ \;\;\;\;\;\;\times 100\%\;-\;strut\;index \end{array}$$

### 2.6. Statistics

A two-way (speed and gait) repeated measures (speed) ANOVA with a Bonferroni adjustment was used to test for differences across speeds and between gaits in all the investigated variables. Statistical analyses were performed using SPSS Statistics (IBM Corp., Version 20.0, Armonk, NY, USA) and the level of significance was set to α = 0.05.

## 3. Results

Significant main effects of speed and gait (and significant interactions) were observed for all the investigated parameters. Since we were not interested in the effect of gait, per se, in the following sections, we do not report the main effect of interaction and the *p* values reported below refer either to the main effect of speed or to the comparison between walking and running at paired speeds.

The estimated walk-to-run transition speed was 7.4 ± 0.2 km/h. Duty factor was unaffected by speed in both locomotor tasks; larger values were reported in walking (about 55%) than in running (about 35%) at all the investigated speeds (see Table 1). The values of maximum dorsi and plantarflexion and the mean values of ankle moment for all the investigated conditions (gait and speed) are reported in Table 1. Significant differences in these parameters were observed as a function of speed both in walking (*p* < 0.01) and running (*p* < 0.05). Larger values of ankle dorsiflexion (*p* < 0.001), plantarflexion (*p* < 0.001) and joint moment (*p* < 0.001) were reported in running than in walking at all the investigated speeds (see Table 1).

The force acting along the MTU (which equals that acting along the AT line of action) was unaffected by walking speed but increased as a function of speed in running. At 5.5 km/h, the AT force was higher in walking than in running (*p* = 0.041), whereas at 6.5 km/h no significant differences were reported between tasks. After 6.5 km/h, AT force was higher in running (*p* = 0.012 and 0.003 for 7.5 and 8.5 km/h, respectively) than in walking (see Table 1).

AT and MTU velocity were affected by walking speed but were unaffected as a function of running speed. At paired speeds, AT and MTU velocity were higher for running than walking (see Table 1).

Even if AT and MTU force are the same quantity, this is not the case for AT and MTU power, because of the differences in shortening velocity. AT and GM MTU power increased as a function of speed both in walking (*p* < 0.001) and in running (*p* < 0.001). In addition, AT and GM MTU power were higher in running than in walking at all the investigated speeds (*p* < 0.001) (see Table 1).

**Figure 2:**
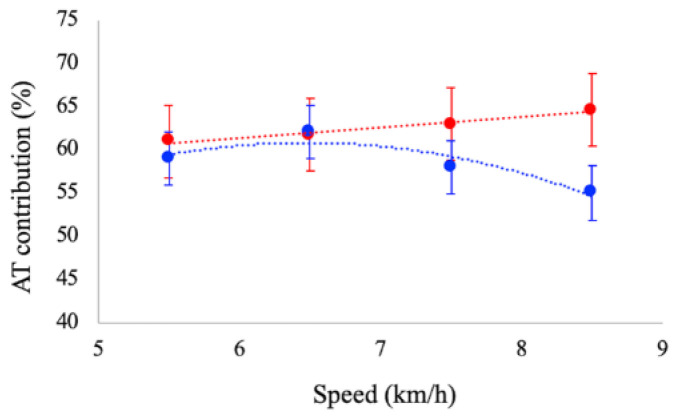
Contribution of the Achilles tendon power to the total mechanical power generated by the GM MTU during walking (blue dots) and running (red dots) at the investigated speeds. Data are means ± SD.

The contribution of the AT mechanical power to the total power provided by the MTU was affected by speed. During running, AT contribution increased as a function of speed (*p* < 0.01), whereas during walking, it tended to decrease (*p* = 0.046) (see Figure 2). At paired speeds, significant differences were reported between tasks at 7.5 and 8.5 km/h: the contribution of AT was significantly larger in running (62% and 64%) than in walking (58% and 55%), respectively.

**Figure 3:**
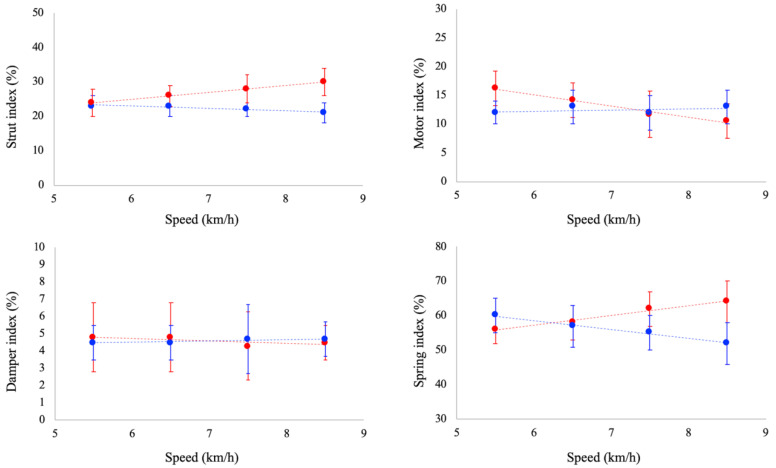
Joint functional indexes (in the stance phase) during walking (blue dots) and running (red dots) at the investigated speeds. Data are means ± SD.

Ankle joint functional indexes are reported in Figure 3. Strut function increased as a function of running speed (*p* < 0.05), whereas it was unaffected by speed in walking. Damper index was unaffected by speed both in walking and running. Motor index decreased as a function of running speed (*p* < 0.05), whereas a slight increase (*p* = 0.052) was observed as a function of speed in walking. Spring index decreased and increased as a function of walking (*p* < 0.05) and running speed (*p* < 0.05), respectively. At paired speed, strut index was significantly larger in running than walking at 6.5, 7.5 and 8.5 km/h. No significant differences between gaits were reported for damper index at all the investigated speeds. Spring index was significantly larger in running at 7.5 and 8.5 km/h, whereas no significant differences were observed between tasks at 5.5 and 6.5 km/h. Finally, motor index was larger in running than in walking at 5.5 km/h, whereas at 8.5 km/h it was larger in walking.

## 4. Discussion

Data reported in this study indicate that Achilles tendon force and ankle spring index are higher in running than in walking at speeds > 7.5 km/h, suggesting that the ankle mechanical behaviour is impaired when walking above the transition speed; this finding provides support for our hypothesis. Indeed, the estimated transition speed (calculated based on Froude’s number of 0.5) was 7.4 ± 0.2 km/h in our subjects, a value close to that reported in the literature for healthy adults: 7.2–7.5 km/h (e.g., Alexander, 1987). We also observed that, in walking, the contribution of the Achilles tendon in determining the total mechanical power at the MTU level tends to decrease at speeds > 7.5 km/h, reinforcing the idea that it is not convenient to walk at these speeds. Hence, our data suggest that the walk-to-run transition could (at least partially) be attributed to the need to preserve AT mechanical behaviour (large values of force and power) and the ankle spring function.

### 4.1. Achilles Tendon Force and Power

From a biomechanical point of view, human walking and running are energy-saving tasks where the spring tissues (e.g., Achilles tendon) store and release elastic strain energy allowing for a reduction of the metabolic demand at the whole-body level [2,21,22,23]. In this study, we focused our attention on the elastic components of the GM MTU as possible determinants of the walk-to-run transition due to their primary role in determining the energy-saving capacity. In a modelling study, Neptune and Sasaki [6] showed that the inability of the plantar flexor muscles to provide large forces during the propulsive phase of walking might be a determinant of the preferred walk-to-run transition speed. Farris and Sawicki [5] confirmed these results in vivo, showing that the gastrocnemius medialis muscle fascicles shortening velocity increases as a function of walking speed, reducing the force capacity of the muscle (in agreement with the F-V relationship). The present data strongly support this idea, since the force acting along the Achilles tendon tends to decrease at walking speeds > 7.5 km/h. Moreover, in agreement with Farris and Sawicki [5], our data showed that switching from walking to running at a speed of about 7.5 km/h restores the force contractile capacity.

A novel finding of this study is in regard to the behaviour of the mechanical power released by the Achilles tendon during walking and running at increasing (and paired) speeds. We observed an increase in AT power as a function of speed both in walking and running and we observed that AT power contribution in determining GM MTU power was different between tasks. During walking and running at 5.5 and 6.5 km/h, AT provides about 60% of the MTU power, with no significant differences between tasks whereas, at higher speeds, the contribution of AT power increases in running and decreases in walking. To our knowledge, no data are reported in the literature regarding the amount of elastic strain energy provided by the Achilles tendon during walking; however, the observation that elastic strain energy increases with speed in running is consistent with the previous literature [19,21,24].

At 7.5 and 8.5 km/h, the energy-saving behaviour of the Achilles tendon is, thus, impaired in walking compared to running; this impairment is expected to be associated with an increase in the mechanical demands of the muscle’s fascicles, leading, ultimately, to an increase in the metabolic demands of walking [8]. Sasaki and Neptune [6], in their modelling study, revealed that running below the transition speed required more muscle fiber work than walking, and, inversely, walking above the transition speed required more muscle fiber work than running. Furthermore, as recently reported by Monte et al. [8], the neuromuscular system is not able to promote “sustainable” GM fascicle mechanics at walking speeds > 7 km/h. This mechanical disadvantage increases the EMG activity required to sustain muscle contraction, increasing the metabolic energy expenditure at the whole-body level.

Therefore, switching from walking to running at a speed of about 7.5 km/h allows for an improvement in the Achilles tendon’s mechanical behaviour, increasing its energy-saving capacity.

### 4.2. Joint Functional Indexes

As previously reported by recent studies [14,15], the joint functional index approach allows for quantifying functional variations of the ankle joint across gaits/speeds.

Even if, so far, no studies have reported values of joint functional indexes during walking and running at increasing (and paired) speed, our data are in line with other observations/results reported in the literature. As an example, Lai et al. [14] investigated the functional indexes of the lower limb joints during walking (at 1.4 m/s) and running (at 4 m/s) and observed a greater spring-like function in running compared to walking, which is compatible with the different dynamics of these motor tasks: mass-spring dynamics of running [25,26] and inverted-pendulum dynamics of walking [27]. In addition, the greater strut-like function in running observed in this study is compatible with in vivo studies that observed a quasi-isometric behaviour of the muscle fascicles in this motor task, while the elastic elements accompany the largest part of the MTU changes in length, thus allowing the ankle to operate mainly as a spring [14,21,28,29,30].

We also observed differences in terms of strut function between tasks as a function of speed, which suggests that during walking at speeds > 5.5 km/h the behaviour of the muscle fascicles could be impaired compared to running. This speculation is supported by a recent study of Monte et al. [8], who observed that the operating length of the GM muscle fascicle is, indeed, impaired at high walking speeds.

The changes in the spring and motor indexes constitute a novel and interesting result. Whereas the spring index was similar between tasks at 5.5 and 6.5 km/h, it was higher in running at 7.5 and 8.5 km/h, suggesting that at speeds > 7.5 km/h the mechanical capacity of the ankle to operate as a spring is impaired when walking. On the other hand, a quasi-opposite trend was observed for the motor index, suggesting that running at slow speeds could not be mechanically advantageous compared to walking. Therefore, the functional indexes suggest that switching from walking to running at a speed of about 7.5 km/h increases the spring-like function of the ankle joint, increasing its the energy-saving capacity.

## 5. Limitations

We did not directly measure the force generated by the GM MTU during walking and running. Instead, we estimated the force acting along the Achilles tendon line of action using an inverse dynamic approach. We then assumed that the GM MTU force is equal to the Achilles tendon force. This approach has been, however, utilized by several authors [5,19,31].

We placed the ultrasound probe on the GM MTJ, which represents the longest portion of the Achilles tendon inside the plantar flexors. Placing the probe on the other MTJs of the triceps surae could, possibly, lead to different results. Indeed, our approach neglects the effects of the inter-muscular force transmission between the individual muscles comprising the ankle plantar flexor group. However, this approach is utilized by several authors [31,32,33].

## 6. Conclusions

Our results suggest that the walk-to-run transition could be partially explained by the need to preserve the spring-like function of the ankle joint. In addition, when walking at speeds faster than the typical transition speed (7.2–7.5 km/h), the Achilles tendon mechanical behavior is impaired: the force acting along its line of action is reduced, as well as its contribution in determining the total mechanical power of the muscle–tendon unit.

## Figures and Tables

**Table 1:** Kinematic and kinetic parameters during walking and running at the investigated speeds. The degrees of ankle dorsiflexion and plantarflexion refer to the maximal values during the stance phase. Ankle moment values correspond to the average value during the entire stance phase. AT force and AT power, as well as gastrocnemius medialis MTU power, are calculated during the propulsive phase only.

	Duty Factor (%)	Ankle Dorsiflexion (°)	Ankle Plantarflexion (°)	Ankle Moment (Nm)	AT Velocity (cm/s)	MTU Velocity (cm/s)	AT Force (N)	AT Power (W)	MTU Power (W)
**Walking**									
5.5 (km/h)	56 ± 4	9.8 ± 2.6	5.4 ± 2.3	0.65 ± 0.22	3.87 ± 0.42	6.56 ± 0.33	1275 ± 96	49.4 ± 20	83.7 ± 27
6.5 (km/h)	55 ± 3	9.9 ± 2.7	6.3 ± 3.3	0.71 ± 0.25	4.9 ± 0.31	7.91 ± 0.35	1327 ± 101	65.2 ± 24	105.3 ± 30
7.5 (km/h)	55 ± 4	10.2 ± 2.8	7.3 ± 3.1	0.76 ± 0.31	7.73 ± 0.42	13.3 ± 0.76	1247 ± 109	96.4 ± 25	166.1 ± 32
8.5 (km/h)	54 ± 3	11.2 ± 3.2	8.8 ± 3.5	0.83 ± 0.29	9.84 ± 0.42	17.9 ± 0.72	1234 ± 106	121.4 ± 27	220.8 ± 37
**Running**									
5.5 (km/h)	35 ± 3 *	15.3 ± 4.3 *	16.5 ± 4.8 *	1.98 ± 1.01 *	15.5 ± 0.84 *	25.5 ± 1.23 *	1127 ± 96 ^	174.9 ± 30 *	286.8 ± 36 *
6.5 (km/h)	36 ± 4 *	16.2 ± 3.6 *	16.9 ± 5.1 *	2.32 ± 1.11 *	16.5 ± 0.93 *	26.7 ± 1.34 *	1211 ± 103	199.6 ± 32 *	323.3 ± 33 *
7.5 (km/h)	35 ± 4 *	17.4 ± 4.1 *	17.7 ± 4.9 *	2.45 ± 1.03 *	16.6 ± 0.87 *	26.4 ± 1.44 *	1356 ± 98	224.8 ± 35 *	357.4 ± 37 *
8.5 (km/h)	37 ± 4 *	17.9 ± 4.0 *	18.1 ± 5.2 *	2.77 ± 1.13 *	16.5 ± 0.99 *	25.5 ± 1.56 *	1415 ± 110 ^	232.9 ± 37 *	360.9 ± 38 *

Footnote: ^ Significant difference (*p* < 0.05) between walking and running at paired speed. * Significant difference (*p* < 0.001) between walking and running at paired speed.

## Data Availability

The raw data supporting the conclusions of this article will be made available by the authors, without undue reservation.
